# Urticaria—Its Treatment by Belladonna

**Published:** 1880-01

**Authors:** 


Urticaria—Its Treatment by Belladonna.—Dr. Q. C. Smith writes us on this subject: "Without stopping to examine the records of medicine, to learn whether or not our use of this valuable remedy in this disease, Belladonna in Urticaria, is original with us, we would note the result of our own experience with it, and add: We do not recollect having seen or heard of it being thus used by any one else. To be brief and to the point: first give a full emetic dose of ipecac, and after it has acted thoroughly, give fl. ext. of belladonna, in small doses, every two hours, until its characteristic flush of the skin is produced on the face, or until vision is considerably disturbed. This degree of impression should be maintained, gradually diminishing the dose, for two or three days. Of course, such constitutional treatment as may be indicated in each case should be duly instituted, though many cases require nothing further than the belladonna.

